# A Hybrid Approach for Improving Image Segmentation: Application to Phenotyping of Wheat Leaves

**DOI:** 10.1371/journal.pone.0168496

**Published:** 2016-12-19

**Authors:** Joshua Chopin, Hamid Laga, Stanley J. Miklavcic

**Affiliations:** 1 Phenomics and Bioinformatics Research Centre, University of South Australia, Mawson Lakes, South Australia, Australia; 2 School of Engineering and Information Technology, Murdoch University, Perth, Western Australia; University of Nottingham, UNITED KINGDOM

## Abstract

In this article we propose a novel tool that takes an initial segmented image and returns a more accurate segmentation that accurately captures sharp features such as leaf tips, twists and axils. Our algorithm utilizes basic a-priori information about the shape of plant leaves and local image orientations to fit active contour models to important plant features that have been missed during the initial segmentation. We compare the performance of our approach with three state-of-the-art segmentation techniques, using three error metrics. The results show that leaf tips are detected with roughly one half of the original error, segmentation accuracy is almost always improved and more than half of the leaf breakages are corrected.

## Introduction

Despite imaging methods for plant phenotyping receiving widespread attention in recent years [1–3], accurate segmentation of plant shoots from images remains a challenging task. In a review of computer vision technologies applied to plant image problems, Lin et al. [4] refer to image segmentation as the single most crucial, open problem. The tips and twists of thin leaves are often overlooked during segmentation, at the expense of not capturing background regions such as soil or shadows [5]. While these small features may at first seem insignificant, they are important for a proper characterization of growth and development. For example, accurately capturing the entire leaf, including tips and twists, provides a more accurate Leaf Area Index (LAI) used to measure relative growth rates of plants [6, 7]. In the application of 3D reconstruction [8–11], from which information on the form and size of a plant can be extracted, failing to capture leaf twists during the segmentation stage will result in a disjointed 3D approximation to the plant. Leaf tips themselves are also essential landmarks for gathering information about the plant. For example, Seginer et al. [12] use the orientation of leaf tips to detect leaf wilting in water stressed plants before any physiological defects occurred. The direction of leaf tips can also be indicative of the amount of light captured and nitrogen accumulated in the leaf [13]. Furthermore, leaf tips are often used to initialize procedures for extracting other plant features. For example, in [14] the authors use leaf tips to fit deformable models for identifying species of weeds in images. Leaf tips are also used as initial starting points in algorithms for measuring individual leaf area [15, 16] and also matching [17, 18] and aligning [19] plants from multiple images.

Techniques for detecting leaf tips and twists in images either make use of a priori information or operate solely on the image in question. Yin et al. [19] match the foreground of the segmented image to a database of leaf templates where locations of leaf tips are known. There are also various techniques operating only on the test image. Tessmer et al. [15] rotate a line about the centre of the plant and label the furthest point of intersection of the line and mask as a corner. Midtiby et al. [16], use the curvature of the segmented foreground region to detect leaf tips and Manh, et al. [14] pass a small moving window over the segmented image and identify features based on the shape of the foreground region inside the window. All of these techniques are directly dependent upon the accuracy of image segmentation. Hence, if leaf tips, twists and axils are not captured during the segmentation stage it will be impossible to locate them accurately using the above techniques. Furthermore, breakages in the segmented plant due to the failure of capturing leaf twists results in spurious corners being detected.

**Fig 1:**
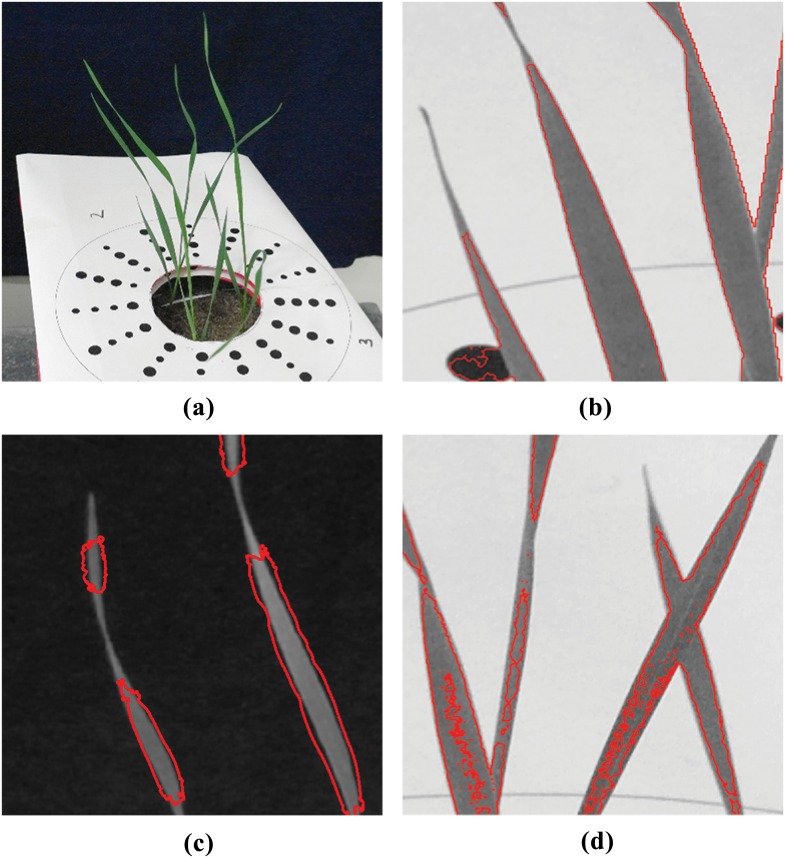
Example results of segmentation using existing techniques applied to wheat leaves. Red boundaries indicate the extent of captured regions. (a) Original image; (b) segmentation by Gaussian Mixture Models in the L*a*b* colour space; (c) segmentation by K-means clustering in the L*a*b* colour space; (d) segmentation by multi-dimensional histogram thresholding in RGB and HSV colour spaces.

Techniques for image segmentation can be broadly classified as spatially guided or spatially blind [20]. Spatially guided approaches make use of colour or intensity of pixels as well as their location and arrangement in an image. Spatially blind techniques disregard the location of pixels and attempt to group or cluster them based only on their colour or intensity. By far the most commonly used segmentation techniques in plant image analysis are spatially blind. The simplest of these approaches is thresholding [21] and is still one of the most commonly used techniques in plant image analysis pipelines [3, 5]. The goal here is to find the threshold that will minimize the intra class variance between pixel intensities higher and lower than the threshold value. Other techniques such as K-means clustering [22], which attempts to minimize the variance of intensity values within a predefined number of clusters, and Gaussian Mixture Models (GMM) [23], which use probability distributions to classify pixels as foreground or background, have also been applied to the plant image analysis problem. However, when stressed, plant leaves can be different in colour, appearing very similar to background regions such as soil or shadows. Hartmann et al. [5] note that capturing stressed regions during segmentation would mean also falsely capturing the background regions. Hence, colours typical to these regions, such as brown and yellow, are excluded during their segmentation stage creating a source of error, especially at plant tips. Examples of inaccuracy during segmentation by GMM, K-Means clustering and thresholding are shown in Fig 1(b), 1(c) and 1(d) respectively.

In this article we propose an algorithm for plant image segmentation that is capable of accurately capturing the entire plant, including leaf tips and twists, even in the thin leaves of plants such as wheat. The procedure, explained in full detail in the Methods section, takes initial inaccurate image segmentations, produced from segmentation techniques such as those named above, and extends them to capture these important features. First the initial segmentation is automatically split at a number of control points, which indicate where leaf tips or twists could reside (Identifying Control Points Section). The algorithm then utilizes the orientation of leaf edges to search for tips and twists which may have been missed during segmentation (Image-informed Evolution of Control Points). Finally, a new control point is anchored at the position of the located features and a deformable model is used to capture the missing parts of the leaf and add them to the segmented image (Attracting Snakes to Image Edges). While the initial segmentation is often created using spatially blind methods, the use of deformable models makes the algorithm spatially guided at these locations. Hence, the method proposed here is a hybrid algorithm, making it less dependent on image colour in regions where image colour has been shown to be a confounding factor. Relying primarily on the direction and magnitude of leaf edges, rather than leaf colour alone, our algorithm is successful over a range of lighting conditions, plant species and plant health levels (Results and Discussion).

## Methods

**Fig 2:**
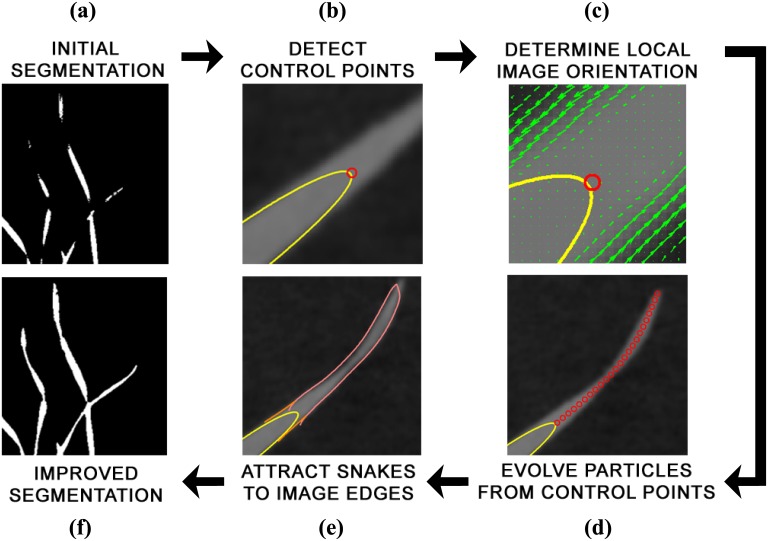
Pipeline of the proposed hybrid method for improving segmentation. Viewed clockwise from top left: (a) The binary image obtained from the initial segmentation; (b) control point detection in segmented leaves by calculating curvature of initial segmentation boundary; (c) orientation field determination in a small window around the control point, which directs the algorithm toward features of interest; (d) evolution of “particles” from an assigned control point to a feature of interest; (e) Insertion and evolution of active contours to capture any features previously missed (f) final result of application of the algorithm, which is an improved segmentation of the plant.

An overview of the algorithm proposed in this chapter is given in Fig 2. The goal of the procedure is to locate regions in a plant image where segmentation may have failed, such as at leaf tips or fine twists in the leaf, and then to improve the initial segmentation. The algorithm begins by taking an inaccurately segmented plant image as input. The segmented image is first dilated and eroded to remove small regions or points of noise. Then, the boundaries, or contours, of the connected components of the segmented image are used to detect control points on the objects. Control points are locations where the initial segmentation has potentially failed to capture an important feature, such as sharp tips, deep axils or twists in a leaf. While they are useful starting points for detecting these features, the control points may reside at a large distance from the actual feature. To resolve this, the local image orientations around the control points are used in a particle evolution stage to direct the algorithm to where the leaf tips, axils or twist should reside. Once the location of the features has been determined, it remains to locate the full boundary of the leaf segment that has not been captured. We use the snakes formulation of Kass et al. [24] to capture the leaf edges near the features of interest, effectively improving the accuracy of segmentation. Since it does not depend on initial segmentation technique, and only requires a binary segmented image as input, our algorithm is afforded a degree of versatility that allows it to be used in a number of plant imaging scenarios. In this section we will outline in detail each stage of the hybrid algorithm.

### Identifying control points

Control points represent locations on the plant where, due to inaccurate initial segmentation, important features such as tips, twists or axils have not been captured. Under the assumption that the shape of the object captured in the segmented image mimics the shape of the true object to a reasonable degree of accuracy, regions of the object near these leaf tips, axils and twists will exhibit high curvature values. By extracting the boundaries of the connected components in the segmented image, we obtain a closed contour that approximates each object and from which we find high curvature regions. In practice, however, the contours created from colour based segmentation are rarely smooth and the noisy curves will cause our algorithm to accept many false positives during the detection of high curvature regions. To address this challenge we smooth each contour individually before approximating gradients. The contours are smoothed using a moving average, weighted by a Gaussian; the procedure is explained in more detail in S1 Appendix. Calculating the curvature at each point on the contour produces a signal whose peaks correspond to the location of high curvature points. We select these high curvature points as control points for the algorithm.

### Image-informed evolution of control points

The control points detected on each contour indicate where the initial segmentation may have failed to capture an important feature. However, depending on the quality of the initial segmentation, the desired feature can reside in a location far from the detected control point. In order to locate each control point’s corresponding feature, we propose a particle evolution procedure.

Our procedure involves identifying the control point as an initial particle and subsequently evolving it in such a way as to follow the local image orientation. The dominant orientations in a small window around the particle should correspond to one or more leaf edges. Algorithm 1 provides an overview of this procedure using pseudo-code. Here, *C* is the total number of contours and *P* is the total number of control points on a given contour. The particle *p* is initialized as the *j*’th control point on the *i*’th contour. The unit vector $\hat{V}=\left(V_{x},V_{y}\right)$ is used to update the particle’s location at each iteration and is initialized as the unit normal, $\hat{n_{p}}$, to the *i*’th contour at the *j*’th control point. The set *t* contains conditions for stopping the particle, where ‘all (*t* ≠ 1)’ indicates that none of the various stopping criteria are currently being met. The direction of $\hat{V}$ in subsequent iterations is taken from the image’s tangent field, *T*, at point *p*. We now give a more in-depth explanation of how $\hat{V}$ and *T* are calculated, as well as other steps of the algorithm.

**Algorithm 1** An overview of the process of image-informed evolution for control points

1: **for** *i* = 1 to *C* **do**

2:  **for** *j* = 1 to *P* **do**

3:   $p=\left(x,y\right),\hat{V}={\hat{n}}_{p}$

4:   **while** all (*t* ≠ 1) **do**

5:    $\hat{V}=\left(V_{x},V_{y}\right)$

6:    $p=p+\hat{V}=\left(x+V_{x},y+V_{y}\right)$

7:   **end while**

8:  **end for**

9: **end for**

#### Step 1

After calculating the normal, ${\hat{n}}_{p}$, of the contour, extra calculations are required to decide whether the control point should evolve inward or outward in the contour’s normal direction. To resolve the ambiguity we consider both cases by adding a trial point at the end of both the inward and outward normals. Again, using the knowledge that the contour’s curvature is high at the control point, the normal direction that evolves the particle so as to add to the length of the contour is the one chosen.

#### Step 2

**Fig 3:**
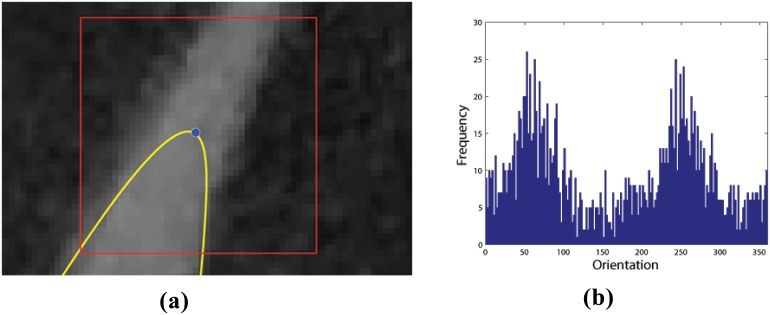
Determining the local orientation field. The figure illustrates the basis for determining the direction of “particle” evolution. (a) A small 40 × 40 pixel window (red) is created with the control point (blue) of the initial contour (yellow) at its geometric centre. (b) A frequency histogram of orientation field angles for every pixel inside the window. As the orientation field of background pixels is for the most part relatively random, the two peaks corresponding to the two leaf edges become prominent in this histogram. These are employed to give direction to the “particle” evolution process.

The particle position should subsequently evolve in a direction $\hat{V}$ corresponding to the image’s local orientation. Fig 3 provides an illustrative example of this process. To calculate the correct orientation, a direction vector $\hat{V}$ is defined at the particle’s current location *p* = (*x*, *y*) based on the direction of local image tangents, taken from the orientation field, *T*, defined as

$$T={\text{tan}}^{-1}\left(\frac{\partial I}{\partial y}/\frac{\partial I}{\partial x}\right),$$ (1)

where partial derivatives are calculated using central difference and single-sided difference at the image edges. The orientation field comprises mostly random orientation vectors in background regions and dominant, informative, orientation vectors at leaf edges. To make use of this fact we introduce a small window of width *w* around the particle’s current location, as depicted in Fig 3(a). The histogram of orientation directions inside the window, based on *T*, is shown in Fig 3(b). Here, the two large peaks at approximately 70 degrees and 250 degrees represent the orientations of opposite leaf edges. After smoothing the histogram, these two peaks correspond to the first and second statistical modes of the distribution, *i.e.*, the first and second most frequently occurring orientations. Note from the example in Fig 3(a) that the tangents on either side of the leaf are in opposite directions. This means that one of the two modes, needs to be reversed in direction to guide the particle’s evolution. The orientation that should be reversed is the one that differs the most from the current contour’s outward pointing normal; this orientation is labeled *θ*_2_. The correct direction and the one to be reversed, *θ*_1_ and *θ*_2_, respectively, determine the new direction vector $\hat{V}$ as

$$\hat{V}=\left(cos\left(\bar{\theta }\right),sin\left(\bar{\theta }\right)\right)$$ (2)

where $\bar{\theta }=\left(\theta_{1}+\theta_{2}+\pi \text{sgn}\left(\theta_{1}-\theta_{2}\right)\right)/2$, sgn(*x*) = ±1, for *x* ≶ 0. For all subsequent iterations the vector $\hat{V}$, derived thus from the previous iteration, is used to determine the correct direction of evolution.

#### Step 3

**Fig 4:**
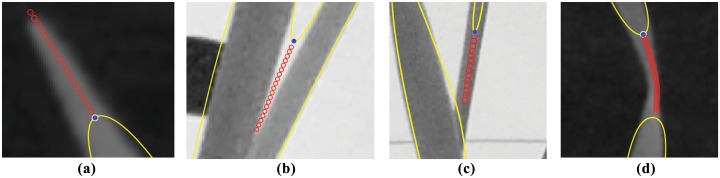
Visual demonstrations of finite-length “particle” evolutions. Four image features, which prove to be problematic to capture in segmentation (leaf tips, leaf axils, leaf joins and leaf twists), lead to different stopping criteria. Particle evolution has stopped in (a) after reaching a leaf corner/tip, in (b) after reaching a leaf axil, in (c) after reaching an intersection of two leaves, and in (d) after passing over a leaf twist.

The penultimate step of this procedure is to update the particle’s position using the unit direction vector $\hat{V}$. This step corresponds to Line 6 of Algorithm 1, where the unit vector $\hat{V}$ is simply added to the point *p*’s current location. Four example evolution paths taken by the particle based on four typical plant leaf scenarios can be seen in Fig 4.

#### Step 4

The preceding steps are repeated until a stopping criterion is satisfied. This step outlines the criteria that are used to stop the particle evolution. The set *t* contains the different criteria for stopping the evolution of the particle. If any of these criteria are met the particle will stop evolving and the algorithm will continue to the next control point. There are different stopping criteria for different plant features, such as leaf tips or twists in the leaf. Below, we will describe each of them in detail.

### Classifying plant features for particle stopping criteria

There are a number of different criteria that could possibly be invoked to cease the particle evolution phase, each one pertains to a different plant image feature. In this work we consider four stopping criteria; leaf tips, leaf axils, leaf edges and leaf twists. Each plant feature contains properties identified by the algorithm and with which a unique stopping criterion for the particle’s evolution can be defined. The set of stopping criteria, *t*, are checked in sequence, according to the order given below.

#### Leaf tip

Let *B*(*θ*) denote the histogram of image orientations, *θ*, inside the moving window, centred on a control point. If the evolving contour reaches a location where the number of points with image orientation *θ*_1_ inside the window is less than some threshold *ϵ*, i.e. |*B*(*θ*_1_)| ≤ *ϵ*, then there is no dominant orientation. This means that the feature is a leaf tip, as shown in Fig 4(a), and the particle is stopped.

#### Leaf axil

If the initial direction of evolution was calculated to follow the inward normal of the contour, then the particle is evolving toward a leaf axil, as in Fig 4(b). This figure shows the typical characteristic that at a leaf axil there are often four leaf edges, two inner and two outer, within the moving window at a leaf axil. The stopping criterion for leaf tips, which uses the frequency of orientations inside the moving window, is no longer suitable since, at a leaf axil, the outside leaf edges still contribute to the frequency of leaf edge orientations.

To overcome this problem we devised a procedure that can distinguish between the inner and outer leaf edges in the vicinity of a leaf axil. A straight line, perpendicular to the particle’s direction of movement, with the same width as the moving window, is constructed. At each point along the line we calculate the dot product between the image orientation field at that point and the direction of the moving particle. Since inside and outside edges of the same leaf have opposite tangent directions, this procedure generates a signal with values approximately +1 and -1 for the inner and outer leaf edges, respectively. Then, it remains only to identify when the signs of the peak or the valley, on the immediate left and right of the evolving point, change. This indicates the absence of the inner leaf edges from the signal, hence, an axil has been reached.

#### Leaf edge

If the evolving particle enters the interior of another contour, at a point which is *not* within a distance *w*/2 of a control point of that contour, two leaves have intersected, as shown in Fig 4(c), and the particle is stopped.

#### Leaf twist

If the evolving particle enters the interior of another contour, at a point which is within a distance *w*/2 of a control point of that contour, the evolving particle has crossed over a twist in the leaf, as shown in Fig 4(d), and the particle is stopped.

### Detecting leaf tips and axils

The control points representing regions where initial segmentation has failed to capture leaf tips, axils, twists and intersections should, after particle evolution, now be within a small radius of their associated features. However, the exact corners of leaf tips and axils are very small features and rarely will the evolving particle terminate exactly on top of them. To detect precisely these important points we utilize knowledge of their approximate location in applications of corner detection.

**Fig 5:**
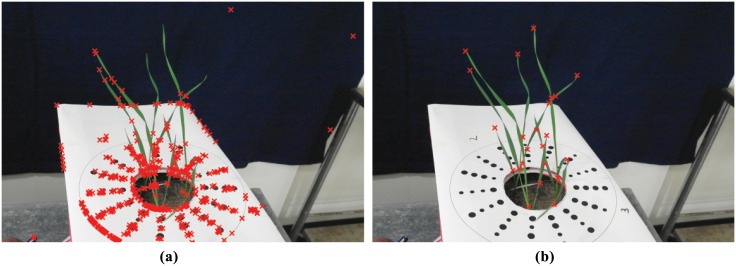
The difference between a global and a local corner detector. (a) Detected corners after running the Harris-Stephens model on the whole image. (b) Detected corners after running the Harris-Stephens model on small windows around terminated particles.

Corner detection is a well-studied problem in image analysis and many potential solutions appear in the literature [25–28]. In our algorithm, we have chosen to use the Harris-Stephens corner detector [26]. The Harris-Stephens corner detector uses the first derivatives of the image to return a value in the corner metric for each pixel in the image. For more information on how the corner metric is calculated, the reader is directed to S1 Appendix. Corner detectors such as the Harris-Stephens model would usually not be suitable for this algorithm due to unrelated image regions returning high values in the corner metric, see Fig 5(a). However, as a result of the evolution of control points, we require the use of only a small search window and knowledge that precisely one corner should exist in that window. As a result, the metric value of the true corner will dominate over results from noise; any unrelated corners will not feature due to the small window size used, see Fig 5(b). We have chosen a window of 40 × 40 pixels for an image of resolution approximately 4500 × 3000 pixels.

### Attracting snakes to image edges

In order to capture the leaf edges near the features of interest we make use of the deformable models proposed by Kass et al. [24]. Their original active contour model is an energy minimization technique for attracting curves to edges in images. Colloquially referred to as snakes, they are used during the last stage of our algorithm to connect the existing contours to plant leaf edges, alongside the path of the contour’s evolved particles. The mathematical formulation of snakes, and how the model has been modified to suit our application, can be found in S1 Appendix.

**Fig 6:**
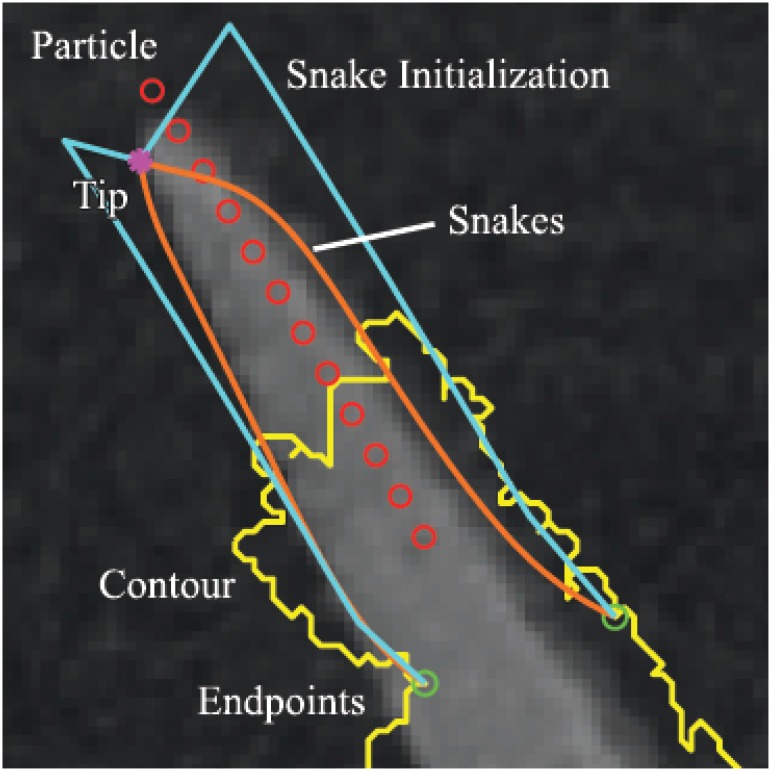
Example of snakes being attracted to leaf edges near a leaf tip. The yellow curve is the boundary of the initial segmented region; red circles denote the path of particle evolution; green circles denote the anchor points for the snakes initialization; magenta star represents a leaf tip; cyan curves are the initial snake contours; and orange curves are the final, evolved snake contours.

An example of a successfully evolved pair of snakes can be seen in Fig 6. Here, the yellow line is the contour from the initial segmentation, the red circles are points along the control point’s evolution path and the magenta star is the leaf tip detected after the particle has stopped. The green circles are the locations of one of the fixed endpoints of each snake, with the second endpoint of each snake being the magenta corner point. The two cyan-blue lines are constructed from a concatenation of a translated particle path passing through the green circle endpoints of the snake, and a straight line segment joining the terminal point of the latter curves to the leaf-tip corner point. These lines are used to initialize the snake. Finally, the two orange curves are the end result of the snake algorithm, attracting the contours to the plant leaf edges. The snake initializations are constructed differently for each of the four distinct plant features discussed throughout this article, based on their different feature classifications. However, in all cases, two endpoints will be fixed to the left and right of the control point, as demonstrated by the green circles in Fig 6.

To calculate where the left, *p*_*L*_, and right, *p*_*R*_, endpoints reside, we again use the curvature signal of the smoothed contour, as defined in Section. If the peaks of this signal correspond to the control points, then the valleys to the left and right of each peak correspond to *p*_*L*_ and *p*_*R*_, respectively. The first points to the left and right of the peak that exhibit a local minimum or, a curvature value less than 0.1, are designated as *p*_*L*_ and *p*_*R*_, respectively.

The distance, *D* = |*p*_*L*_ − *p*_*R*_|, between the endpoints *p*_*L*_ and *p*_*R*_ is used as an approximation of leaf width. As such, the left and right snakes can be initialized orthogonal to the evolved particle’s path, on either side, at a distance of *D*/2. Now that one set of the snakes’ endpoints is fixed and their bodies are initialized, it remains only to fix the opposite set of endpoints. These are unique to the different types of plant features.

- **Leaf tip or axil.** At a leaf tip or axil both endpoints are fixed at the same position. This is the location of the detected leaf tip or axil, denoted by a magenta star in Fig 6.
- **Leaf twist.** At a twist in the leaf the endpoints are fixed at the locations of *p*_*L*_ and *p*_*R*_ on the opposing contour.
- **Leaf intersection** Where one leaf intersects or emerges from another, the two endpoints are fixed either side of where the intersection occurs. *p*_*L*_ and *p*_*R*_ are located at points to the left and right, respectively, of the intersection at a distance of *D*/2.

### Creating the improved segmentation

In the final step of the algorithm a new segmented image is created based on the evolved snakes and the initial segmentation. All plant boundaries, which are the points along the evolved snakes, are superimposed onto a blank image, the same size as the original one, by rounding to the nearest pixel value. To ensure that there are no gaps in the new plant boundaries the new binary image is dilated by one pixel. The boundaries are then filled, to gain a new segmentation, and eroded by one pixel, to account for the previous dilation.

## Results and Discussion

We tested our approach using three different initial segmentation techniques on a range of different types of plant images, three examples are presented here and results for the collection of 15 images, for each type, are presented in S1 Appendix. First, we manually segmented these images and used them as ground truth for evaluating the accuracy of the proposed approach. Three different metrics were used, based on measures of importance in a plant biological context. First, we estimated the Leaf Surface Area (LSA), used for establishing growth rates, from the 2D image using the Sorenson-Dice Index (SDI) [29, 30]. The Sorenson-Dice Index gives a value of one for perfect segmentation and zero when no foregound pixels are captured. Let *S* be the set of points in the foreground of our segmentation and *G* be the set of points in the foreground of the ground truth, then

$$SDI=\frac{2*(S\cap G)}{S+G}.$$

The second metric measures the averge distance, in pixels, from the manually labelled leaf tips to the nearest point in the segmented image. This metric is important when seeking to accurately detect and track leaf tips. Finally, we count the number of leaf breakages in each segmented image i.e. locations where twists in the leaf have not been captured, making a single leaf appear as two disjoint ones. This metric is important for the task of 3D reconstruction and leaf tracking over time.

**Fig 7:**
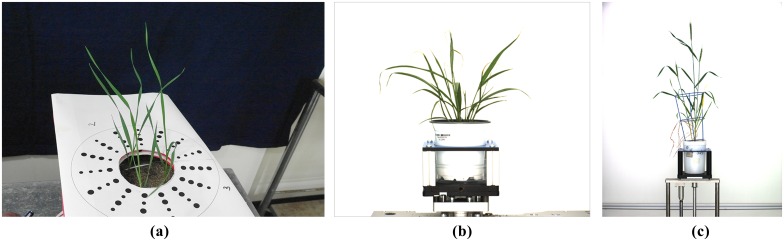
Representatives of the three types of images analysed with the algorithm proposed in this paper. Resulting data for these specific images are given in Table 1. Results of application to four other images of each type can be found in the S1 Appendix file.

**Fig 8:**
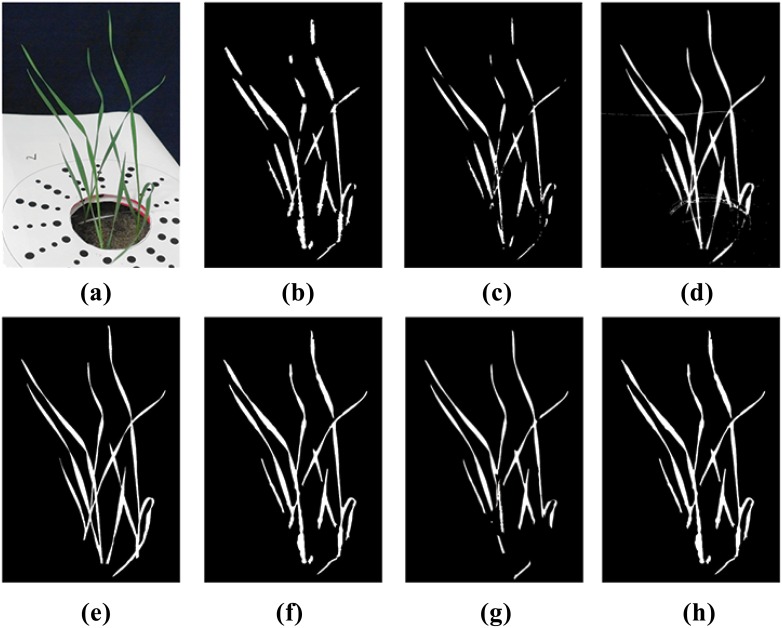
Visual demonstration of improved segmentation using our algorithm applied to sample Image 1. The original image and the manually-obtained ground truth are presented in (a) and (e), respectively. Panels (b) and (f) respectively show initial segmentation using K-means clustering and improved segmentation after application of our algorithm. Panels (c) and (g) respectively show initial segmentation using Multidimensional Histogram Thresholding and improved segmentation after application of our algorithm. Panels (d) and (h) respectively show initial segmentation using Gaussian Mixture Models and improved segmentation after application of our algorithm.

The primary rows of Table 1 present results of our analysis of the three sample images. Within each main row are specific data based on our three chosen measures of accuracy, before and after the application of our hybrid algorithm and subsequent to segmentation attempts using standard techniques of GMM, K-means and MHT (columns 4–6). These particular techniques were chosen as they are used widely throughout the literature and, like the algorithm presented here, are fully automated after setting some initial parameters. Approaches requiring learning, supervision or interaction were avoided as these may effect either the high-throughput analysis or the analysis throughout varying backgrounds. For the images and segmentation techniques studied here, our approach provides an improvement in segmentation accuracy. The specific images 1–3 referred to in Table 1 are shown in Fig 7(a)–7(c), respectively. The results shown in Table 1 are also visualized in Fig 8 for the case of sample Image 1. In summary, our algorithm captured leaf tips with an error that is approximately one half of the original error. By capturing sharp twists in the leaf our algorithm greatly reduces the number of leaf breakages that occurred in the original segmentation. Finally, our algorithm almost always improved the SDI of the initial segmentation, with only a few of the samples resulting in very small SDI reductions. One area where the algorithm can be improved is the evolution step where control points are located in highly noisy regions such as the soil in the pot of Fig 7(a). As Fig 8 shows, in such regions, the degree of improvement is still highly dependent on the accuracy of the original segmentation.

**Table 1:** Results of our method applied to the three example images in Fig 7. Three different measures of accuracy are used: the Sorensen Dice Index (SDI), the average distance to leaf tips (with standard deviation), and the number of leaf breakages. The three initial segmentation methods, listed in Fig 1, are considered. Data includes accuracy measures before and after application of our algorithm for improved segmentation.

			GMM	K-means	MHT
Image 1	SDI	Before After	0.9 0.92	0.80 0.87	0.76 0.85
	Tip distance (px)	Before After	36 (±38) 19 (±12)	89 (±48) 21 (±23)	110 (±54) 34 (±21)
	Breakages	Before After	9 2	17 8	19 7
Image 2	SDI	Before After	0.76 0.90	0.68 0.74	0.6 0.78
	Tip distance (px)	Before After	62 (±59) 38 (±36)	110 (±57) 41 (±40)	96 (±60) 52 (±48)
	Breakages	Before After	14 7	17 8	14 8
Image 3	SDI	Before After	0.84 0.81	0.65 0.74	0.68 0.67
	Tip distance (px)	Before After	16 (±16) 11 (±7)	32 (±19) 18 (±16)	31 (±22) 13 (±13)
	Breakages	Before After	4 4	12 8	18 13

Overall MHT was the worst performing, yet fastest (a detailed report on computation times is given in the following section), of the initial segmentation techniques. Hence, our algorithm was able to provide a 10–15% increase in SDI in many of the test images, as well as greatly decreasing the error in capturing tips and breakages. K-means clustering was more accurate and more time consuming than MHT, but still failed severely in capturing leaf tips. In most cases our algorithm increased the SDI of the k-means segmentation by a small amount but decreased the error in capturing tips by approximately half. Finally, the GMM approach was the most accurate of the three initial segmentation techniques and also by far the most time consuming. Our approach provided no significant difference in SDI, yet was still able to significantly decrease the error in capturing leaf tips and breakages.

**Fig 9:**
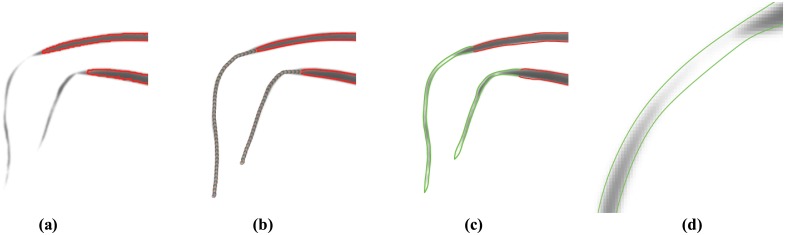
Visual demonstration of improved feature capture of leaves exhibiting sharp twists. (a) A region of the plant in sample Image 2 not properly segmented using K-means clustering (boundary of segmented region appears in red). (b) Overlaid paths of evolving particles beginning at control points and ending at leaf tips. (c) Final converged snake contours (in green) initialized using the particle paths and then attracted to leaf edges. It is clear that the full lengths of the leaves have now been captured. (d) An expanded view of a section of the newly captured plant region containing a leaf twist, illustrating the algorithm’s ability to capture very faint plant leaves.

**Fig 10:**
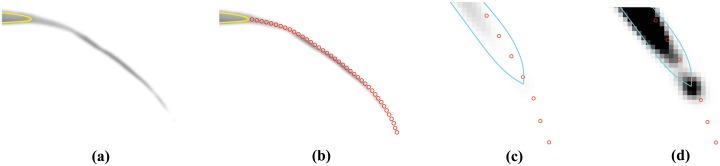
Visual demonstration of improved feature capture of very faint leaf tips. (a) A region of the plant in sample Image 2 not properly segmented using K-means clustering (boundary of segmented region appears in yellow). (b) The path taken by an evolving particle, beginning at a designated control point and ending at a leaf tip. (c) An expanded view of the leaf tip region with the converged snake (in blue) anchored at the detected leaf tip. (d) The same image with image contrast increased to highlight the level of detail finally captured.

We demonstrate in Figs 9 and 10 the accuracy of our algorithm in detecting challenging leaf features. Fig 9(a) shows an enlarged region of an image with two leaves containing twists, that have not been completely captured during the initial segmentation. In Fig 9(b) the evolving particles of our algorithm were initialized at the detected control points, evolved past these sharp twists and finally stopped at the leaf tip. Fig 9(c) and 9(d) show the converged snakes, which have been initialized using the path of the evolved particles and then used to capture the originally undetected leaf regions. Note that where twists in the leaf occur there are very few pixels of leaf material, yet the algorithm still evolves the particle over these regions. Fig 10 further emphasises this point, showing a captured leaf tip that was originally not clearly visible to the human eye. Fig 10(a) shows a leaf tip not being captured by the MHT algorithm and Fig 10(b) shows our algorithm evolving a particle towards the missed tip. In Fig 10(c), the leaf tip has been detected and snakes were evolved to capture the missing part of the leaf. From a cursory glance it would appear as though the algorithm has failed and captured part of the background. However, after increasing the contrast of the same image, Fig 10(d), we see that the snakes have anchored at an actual leaf tip and been attracted to faint leaf edges.

### Computation time

The most time consuming part of our algorithm is evolving the active contours. Hence, the computation time required is proportional to image resolution and the number of control points. Using a machine with an Intel Core i7-2720QM CPU @220GHz processor and 8 gigabytes of RAM, using our algorithm on the three sample images used in this study, of resolution 4500 × 3000, 2500 × 2000 and 2000 × 2500, took, respectively, an average of 60 seconds, 20 seconds and 18 seconds, over the three different initial segmentation techniques. In contrast, the MHT approach took approximately two seconds, K-means took approximately 25 seconds and GMM took more than two hours to segment the images.

### Parameter selection

Our algorithm requires the setting of a number of image resolution-dependent parameters as input. However, the parameters are mostly robust and often intuitive to select. For all images studied in this chapter, if the resolutions of two images were approximately the same, so too were their parameter values. Here, we provide an overview of each of the parameters, the values chosen for the three sample images are given in Table 2.

**Table 2:** Parameters of the proposed algorithm. *m* is the width of the mask used for image dilation and erosion, *r* is the radius of the contour smoother, *w* is the width of the moving window during particle evolution, *σ* is the variance of the Gaussian smoother and *α* and *λ* are weighting coefficients for the snakes model.

	*m*	*r*	*w*	*σ*	*α*	*λ*
Image 1	5	25	40	2	10	10000
Image 2	5	10	20	1	7	1000
Image 3	5	10	20	1	7	1000

#### Erosion and dilation

The first parameter to select is the width, *m*, of the square mask used for dilating and eroding the segmented image. As this step is purely for removing small areas of noise, the same size was used for the mask in all images analysed here.

#### Radius of contour smoother

The next parameter to select is *r*, the radius of the smoother applied to the contours of plants. Since appropriate values of *r* will depend on how densely populated the contour points are, and hence how high the resolution of the image is, these values are intuitive to select. Under-smoothing the contours will result in spurious control points, but due to the various stopping criteria for evolving particles, the control points will be quickly discarded.

#### Window size

Another important parameter is the width, *w*, of the window used for evolving control points toward leaf tips. This parameter is dependent upon image resolution and leaf width. The user should ensure that the window is large enough to contain a sufficient amount of background pixels but small enough so as to not capture other leaves.

#### Gaussian smoother

The variance, *σ*, of the Gaussian applied to the image determines the degree to which the image is smoothed. The parameter *σ* is important because it plays a pivotal role in determining the orientations calculated during the particle evolution stage. Images with higher resolution require larger values of *σ*. Furthermore, over-smoothing is preferable to under-smoothing as long as the leaf is still visible. Especially in high resolution images, a lack of smoothing can lead to noise influencing the orientation calculations.

#### Snakes parameters

The two parameters that require tuning are *α* and *λ*, which attempt to minimize the length of the snake and attract it to image edges, respectively. Parameters for the snakes model are often chosen on a trial and error basis and depend upon object shape [31] and texture [32]. However, as a rule of thumb, for high resolution images values of *λ* are often larger and values of *α* often smaller.

## Summary Remarks

To summarize, the algorithm proposed in this article is an automated tool for improving the segmentation accuracy of plant images. Many existing techniques fail to capture small, yet important, image details of the plant, such as leaf twists or tips. Our algorithm is not only capable of capturing these features but also mending breakages that occur in leaf images when using an inaccurate initial segmentation. For the sample images chosen here our algorithm reduced the average error of leaf tip detection by approximately half and almost always provided an increase in accuracy for leaf surface area estimation. We have shown that the improvements achieved by our algorithm can assist with LAI estimation, leaf tip detection, tracking and tracing, and we postulate that it can also improve the accuracy of multi-view 3D reconstruction. Furthermore, after setting a few initial parameters the method is fully automated, requiring no user input. A few limitations were discovered during the analysis of the algorithm. Firstly, plants in regions of highly inhomogeneous backgrounds, such as coarse soil, suffered from lower increases in accuracy. This occurs when image gradients of the background interfere with plant leaf orientation calculations. Also, the parameter for choosing the size of the moving window was somewhat sensitive. As this parameter uses leaf width in pixels, it is dependent upon both plant leaf morphology and image resolution. During initial testing of the algorithm a few tries were required to determine correct values for this parameter.

One potential direction of future work is to reduce the number of parameters required all together, through either machine learning or further image analysis techniques. Another avenue for future work is to develop an alternative approach for determining local leaf edge orientation in noisy image regions such as those sections containing soil. Still on the subject of plant phenotyping, it is natural, and probably of wider interest, to consider the application of this method to the analysis of images of roots, either as 2D scans or as camera images of plant roots grown in transparent media. In either case, to achieve an accurate classification and quantification of root features [33, 34], it is critically important to completely capture root tips (particularly so in the case of [33], which relies on root tip properties to distinguish between primary and lateral roots). Although it may not be of great benefit for such goals as LAI estimation (available segmentation procedures do sufficiently well here), a more accurate segmentation of broad leaf plants using the algorithm proposed here, to capture sharp tips and edge protrusions and edge depressions, may be of some significance for the purpose of leaf species classification [17, 18]. Finally, we point out this algorithm can be applied to improve the segmentation of images exhibiting other objects besides plants but with sharp features.

## Supporting Information

S1 Appendix Analysis details and supplementary results. (PDF)
