# Supplementary material for: A Hybrid Approach for Improving Image Segmentation: Application to Phenotyping of Wheat Leaves
Source: PLoS One. 2016 Dec 19;11(12):e0168496. doi: 10.1371/journal.pone.0168496 (PMC5167398; doi:10.1371/journal.pone.0168496)
Supplement: S1 Appendix — (PDF) [file pone.0168496.s001.pdf]

A Hybrid Approach for Improving Image Segmentation: Application to Phenotyping of Wheat Leaves

Joshua Chopin, Hamid Laga, Stanley J Miklavcic

S1 Appendix

Curvature calculations for control point detection

Let C be the initial contour and C_s be a smoothed version of the contour, then

$$C_s^i = \omega_r \sum_{k=-r}^{k=r} C^{i+k} \tag{S1}$$

where $i = 1 : N$ are the N points of the contour, r is the width of the moving average and ω_r is a one dimensional gaussian window of width r. Finally, the curvature, κ_i^j, of contour j at point i is calculated as

$$\kappa_i^j = \frac{|(x_i^j)'(y_i^j)'' - (y_i^j)'(x_i^j)''|}{((x_i^j)'^2 + (y_i^j)'^2)^{\frac{3}{2}}} \tag{S2}$$

where the derivatives are calculated using a fourth order approximation, *i.e.*

$$(x_i^j)' = \frac{1}{12}(-x_{i+2}^j + 8x_{i+1}^j - 8x_{i-1}^j + x_{i-2}^j). \tag{S3}$$

Calculating the corner metric

Firstly we compute the second-moment matrix, or structure tensor, which is given as

$$A = \begin{pmatrix} I_x^2 & I_x I_y \\ I_x I_y & I_y^2 \end{pmatrix}, \tag{S4}$$

where I_x and I_y are the derivatives of the image in the x and y direction respectively. Then the value returned by the corner detector, H is

$$H = \det(A) - \kappa \cdot \mathrm{trace}(A). \tag{S5}$$

Mathematical formulation of snakes

The original formulation of snakes states that for a planar curve parameterized by arc length $C : [0,1] \to \mathbb{R}^2, q \mapsto (x(q), y(q))$, the following energy should be minimized,

$$\begin{aligned} E(C) = \alpha \int_0^1 |C'(q)|^2 dq \; + \; \beta \int_0^1 |C''(q)|^2 dq \\ - \lambda \int_0^1 |\nabla I_G(C(q))|^2 dq, \end{aligned} \tag{S6}$$

where $\nabla I_G = (\nabla I * G)$ is the gradient of the image I, smoothed with a Gaussian kernel of parameter σ and the weights α, β and λ are real positive scalar parameters that define the importance of each of the three terms.

To numerically solve the functional in Equation S6 we again follow the approach of Kass et. al [1] who use the following pair of evolution equations

$$\begin{aligned} \mathbf{x}_t &= (\mathbf{A} + \gamma \mathbf{I})^{-1}(\gamma \mathbf{x}_{t-1} - \lambda \mathbf{f}_{\mathbf{x}}(\mathbf{x}_{t-1}, \mathbf{y}_{t-1})) \\ \mathbf{y}_t &= (\mathbf{A} + \gamma \mathbf{I})^{-1}(\gamma \mathbf{y}_{t-1} - \lambda \mathbf{f}_{\mathbf{x}}(\mathbf{x}_{t-1}, \mathbf{y}_{t-1})), \end{aligned} \tag{S7}$$

where bolded variables represent matrices or vectors. Here $(\mathbf{x}_t, \mathbf{y}_t)$ are vectors containing the x and y coordinates of the contour at time t, $\mathbf{A}$ is a tridiagonal matrix containing the parameter α and is used for approximating contour derivatives. It is defined as follows,

$$A(i,j) = \begin{cases} 2\alpha & \text{if } i = j \\ -\alpha & \text{if } i = (j-1) \text{ or } (j+1) \\ 0 & \text{otherwise} \end{cases} \tag{S8}$$

γ is a step size, $\mathbf{I}$ is an identity matrix the same size as $\mathbf{A}$ and $\mathbf{f_x}$ and $\mathbf{f_y}$ are matrices containing the x and y partial derivatives of the external vector field, respectively.

The snakes formulation seeks to minimize the energy defined above by finding a trade-off between contour smoothness and distance to image edges. As a result of the contour being constrained to smoothness, sharp corners are often rounded off and not captured. To modify this model for evolving open arcs with fixed endpoints we simply

change the matrix A to

$$A(i,j) = \begin{cases} 1 & \text{if } i = 1 \text{ or } j = N \\ 2\alpha & \text{if } i = j \\ -\alpha & \text{if } i = (j-1) \text{ or } (j+1) \\ 0 & \text{otherwise.} \end{cases} \tag{S9}$$

Where each open snake will correspond to a leaf edge, and the fixed endpoints will correspond to leaf features such as corners, axils, or twists in the leaf.

Results of the algorithm applied to a larger data set

The following tables give results of analyses of 4 further images of each of the types represented in Figure 7 of the paper.

		GMM	K-means	MHT			GMM	K-means	MHT
SDI	Before	0.87	0.77	0.70	SDI	Before	0.79	0.73	0.63
	After	0.88	0.77	0.75		After	0.81	0.80	0.74
Tip distance	Before	53 (±58)	137 (±124)	112 (±137)	Tip d	Before	22 (±13)	124 (±156)	131 (±169)
(px)	After	11 (±15)	67 (±59)	67 (± 75)	(px)	After	16 (±11)	78 (±101)	81 (± 95)
Breakages	Before	8	13	13	Breakages	Before	8	13	14
	After	3	4	7		After	3	4	4

		GMM	K-means	MHT			GMM	K-means	MHT
SDI	Before	0.79	0.77	0.49	SDI	Before	0.71	0.77	0.54
	After	0.84	0.84	0.61		After	0.68	0.77	0.68
Tip distance	Before	39 (±44)	73 (±53)	145 (±216)	Tip distance	Before	66 (±57)	60 (±51)	315 (±262)
(px)	After	14 (±15)	18 (±23)	48 (±79)	(px)	After	49 (±31)	49 (±32)	121 (±164)
Breakages	Before	11	11	17	Breakages	Before	8	10	17
	After	4	6	9		After	6	6	11

S1 Table. Our algorithm applied to four images of type 1: plant groups with a complex background (Figure 7(a)).

		GMM	K-means	MHT			GMM	K-means	MHT
SDI	Before	0.54	0.88	0.73	SDI	Before	0.86	0.93	0.65
	After	0.56	0.87	0.76		After	0.88	0.92	0.73
Tip distance	Before	137 (±48)	97 (±58)	135 (±37)	Tip distance	Before	50 (±48)	31 (±30)	96 (±105)
(px)	After	111 (±45)	52 (±19)	73 (±35)	(px)	After	19 (±23)	11 (±16)	59 (±72)
Breakages	Before	3	3	2	Breakages	Before	3	3	7
	After	0	0	1		After	1	1	3

		GMM	K-means	MHT			GMM	K-means	MHT
SDI	Before	0.94	0.92	0.80	SDI	Before	0.74	0.87	0.68
	After	0.93	0.92	0.84		After	0.82	0.89	0.79
Tip distance	Before	37 (±23)	28 (±38)	56 (±54)	Tip distance	Before	77 (±77)	24 (±20)	86 (±82)
(px)	After	13 (±9)	13 (±15)	21 (± 24)	(px)	After	41 (± 48)	19 (±19)	53 (±46)
Breakages	Before	4	5	7	Breakages	Before	11	13	19
	After	0	2	2		After	5	4	10

S2 Table. Our algorithm applied to four images of type 2: individual plants without frame support (Figure 7(b)).

		GMM	K-means	MHT
SDI	Before	0.7	0.78	0.62
	After	0.76	0.84	0.68
Tip distance (px)	Before	57 (±45)	33 (±31)	45 (±28)
	After	36 (±19)	24 (±15)	29 (±16)
Breakages	Before	6	9	13
	After	1	3	3

		GMM	K-means	MHT
SDI	Before	0.88	0.51	0.63
	After	0.91	0.69	0.74
Tip distance (px)	Before	13 (±10)	73 (±50)	68 (±34)
	After	9 (±10)	24 (± 29)	29 (±21)
Breakages	Before	4	5	9
	After	0	0	2

		GMM	K-means	MHT
SDI	Before	0.87	0.69	0.66
	After	0.87	0.71	74
Tip distance (px)	Before	8 (±5)	32 (±27)	44 (±25)
	After	7 (±3)	17 (±18)	19 (±23)
Breakages	Before	2	14	12
	After	2	6	8

		GMM	K-means	MHT
SDI	Before	0.78	0.77	0.63
	After	0.81	0.82	0.70
Tip distance (px)	Before	27 (±30)	33 (±51)	71 (±98)
	After	11 (±19)	19 (±30)	50 (±55)
Breakages	Before	7	11	14
	After	4	4	6

S3 Table. Our algorithm applied to four images of type 3: individual plants with frame support (Figure 7(c)).

References

1. Kass M, Witkin A, Terzopoulos D. Snakes: Active contour models. International Journal of Computer Vision. 1988;1(4):321–331.
